# Evaluation of the learning curve and complications in unilateral biportal endoscopic transforaminal lumbar interbody fusion: cumulative sum analysis and risk-adjusted cumulative sum analysis

**DOI:** 10.1186/s13018-024-04674-3

**Published:** 2024-03-21

**Authors:** Wenlong Guo, Jingyao Ye, Tong Li, Yang Yu, Xiaohong Fan

**Affiliations:** 1 Department of Clinical Medicine, Chengdu University of Traditional Chinese Medicine, Chengdu, 610000 China; 2 Department of Orthopaedics, Hospital of Chengdu University of Traditional Chinese Medicine, Chengdu, 610075 China

**Keywords:** Learning curve, Cumulative sum, Risk-adjusted cumulative sum, Unilateral biportal endoscopic, Lumbar interbody fusion

## Abstract

**Purpose:**

To evaluate the learning curve and complications in unilateral biportal endoscopic transforaminal lumbar interbody fusion (ULIF) using the Cumulative Sum (*CUSUM*) analysis and Risk-adjusted Cumulative Sum (*RA-CUSUM*) analysis.

**Methods:**

This study retrospectively analyzed 184 consecutive patients who received ULIF in our hospital, including 104 males and 80 females. *CUSUM* analysis and *RA-CUSUM* analysis were used to evaluate the learning curve of ULIF based on the operation time and surgical failure rate, respectively. All postoperative complications were defined as surgical failure. Variables of different phases were compared based on the learning curve.

**Results:**

The *CUSUM* analysis showed the cutoff point for ULIF was 29 cases, and the *RA-CUSUM* analysis showed the cutoff point for ULIF was 41 cases**.** Operating time and hospital stay were significantly decreased as the learning curve progressed (*P* < 0.05). Visual analogue score (VAS) and Oswestry disability index (ODI) at the last follow-up were significantly lower than preoperatively. At the last follow-up, a total of 171 patients reached intervertebral fusion, with a fusion rate of 92.9% (171/184). A total of eleven complications were observed, and *RA-CUSUM* analysis showed that the incidence of complications in the early phase was 17.07% and in the late phase was 2.6%, with a significant difference (*P* < 0.05).

**Conclusion:**

ULIF is an effective minimally invasive lumbar fusion surgical technique. But a learning curve of at least 29 cases will be required to master ULIF, while 41 cases will be required to achieve a stable surgical success rate.

## Background

The development of the spinal endoscopic techniques and innovations in instrumentation have enabled spine surgeons to achieve decompression of the spinal canal and even interbody fusion through indirect visualization with the assistance of spinal endoscopy [1]. Unilateral biportal endoscopy (UBE) is an emerging minimally invasive spinal endoscopy technique that has attracted the attention of spine surgeons for its advantages of less blood loss, less trauma, and faster postoperative recovery [2, 3]. Since Heo applied the UBE technique to transforaminal lumbar interbody fusion (TLIF) for the first time and got satisfactory outcomes in 2017 [4], the indications for UBE have gradually expanded to various degenerative spinal diseases. Studies have demonstrated that ULIF has shown favorable clinical outcomes in the treatment of lumbar degenerative diseases [5]. However, ULIF remains challenging in its early implementation. Spine surgeons are eager to master the technique, which requires recommendations and references, especially on how to overcome the learning curve.

Although studies have been performed to describe the learning curve of UBE, these studies focused on the decompression of the UBE technique, and almost no studies focused on the learning curve of ULIF. Compared to decompression alone, ULIF seems more complicated and challenging. Therefore, the purpose of this study is to analyze the learning curve of ULIF through *CUSUM* analysis based on operation time and *RA-CUSUM* analysis based on surgical failure rate [6, 7] to offer quantitative evidence for determining the optimal repetition number for mastering ULIF.

## Methods and materials

### Participants

We performed a single-center, retrospective study that reviewed the consecutive patients who underwent ULIF in the Department of Orthopaedics of our hospital from September 2019 to August 2022. All operations were performed by the same surgeon who had moderate experience in UBE (no less than 150 cases) and open lumbar fusion surgery but had never performed minimally invasive lumbar fusion surgery.

This study protocol was approved by the Ethics Committee of Chengdu University of Traditional Chinese Medicine. As the current study was retrospective in nature and data analysis was performed anonymously, this study was exempt from requiring informed consent from patients. There was no treatment other than that routinely implemented during hospitalization, as well as no additional risk for the patients involved.

Inclusion criteria were as follows: (1) patients with low back pain or radicular leg pain, with or without intermittent neurological claudication; Computed Tomography (CT) or Magnetic Resonance Imaging (MRI) showed lumbar spondylolisthesis (meyerding grade I or II), lumbar spine instability or lumbar spinal stenosis; (2) the absence of improvement after conservative treatment for at least three months; (3) the clinical data were complete; and (4) follow-up were not less than six months.

Exclusion criteria were as follows: (1) Lumbar tuberculosis, tumor, infection, or trauma; (2) osteoporosis, T value less than–2.5 [8]; (3) more than two surgical levels; (4) prior lumbar surgery.

### Surgical procedures

**Fig. 1:**
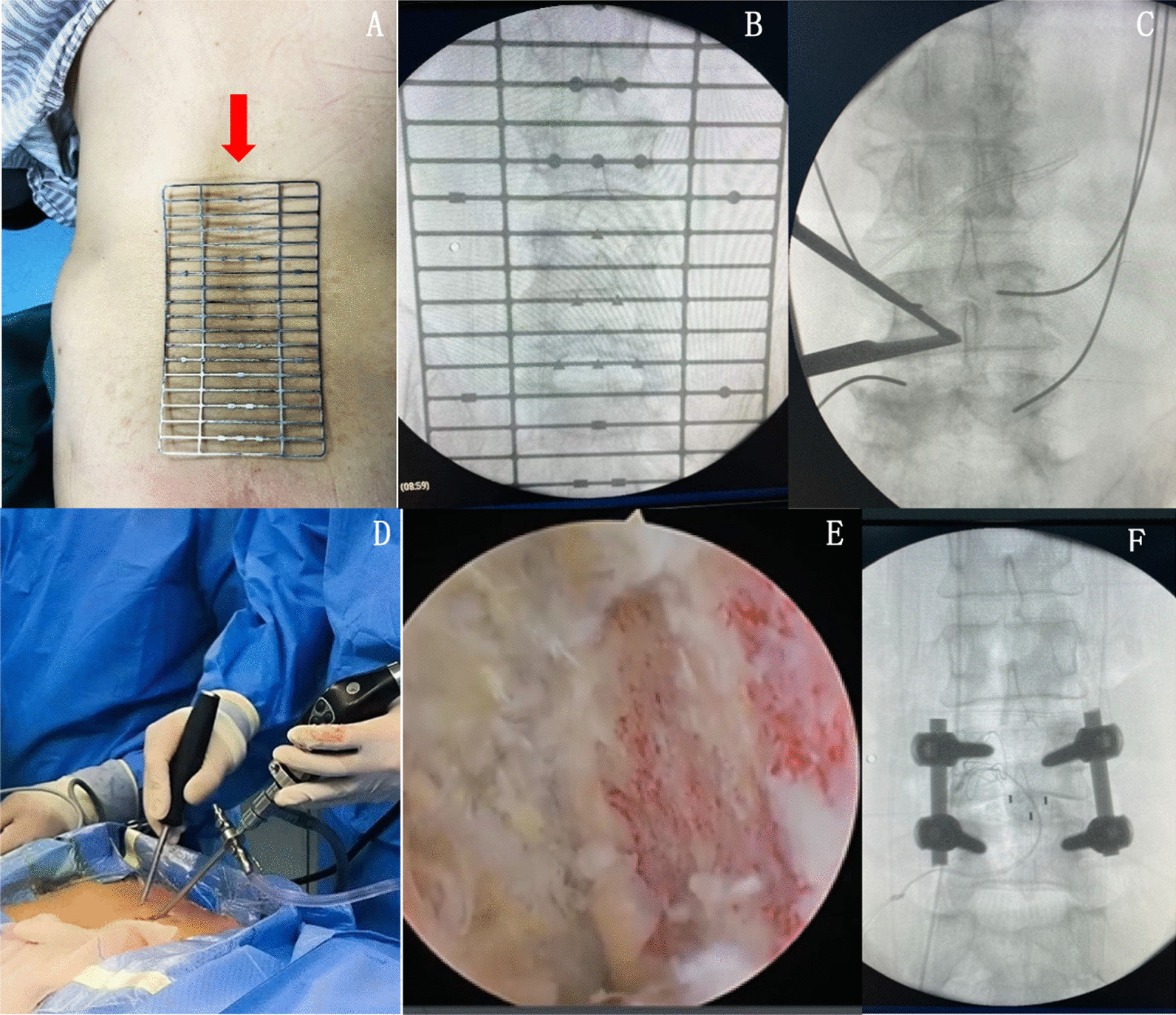
**A-B** Fluoroscopy under the C-arm (The red arrow points to a special localization plate used in spinal surgery at our center.) **C** Use the C-arm to reconfirm the surgical site after surgical channels establishment **D** Surgical area **E** Preparation of endplates under endoscopic monitoring **F** Placement of four pedicle screws

The patient was placed in a prone position under general anesthesia with the abdomen suspended. After routine disinfection, using C-arm fluoroscopy to identify the target vertebra, marking the insertion point of the vertebral pedicle, then inserting guide wires percutaneously along the pedicle. Take the right approach for example, two oblique incisions were made about 1.5 cm from the midline of the spine at the lower edge of the upper endplate and the upper edge of the lower endplate. The lower incision served as the viewing channel, whereas the upper incision served as the working channel. The saline was suspended at a height of approximately 50–60 cm from the incision and connected to a 30° arthroscope. A serial tubular dilator gradually expands the incision and subcutaneous tissue. Then using osteotome and grinding drill to remove the inferior articular process from inside to outside, and then removed the superior articular process, the excised lamina and articular process were used as autologous bone. Remove part of the ligamentum flavum to expose the intervertebral disc. Any tissue compressing the spinal cord and nerve roots was removed. Then remove the overlying cartilage and preserve the hard subchondral bone to prepare upper and lower endplates. Endoscopic insertion of the intervertebral space confirmed that the endplate cartilage had been removed. Cage tryout was done to determine cage size. A cage filled with autologous bone is placed between vertebrae under fluoroscopic and endoscopic guidance. Allogeneic or autologous bone is compressed around the cage. The decompression of the spinal canal was checked to clean up the occult compression and the radiofrequency probe was used for hemostasis after confirming complete decompression. After this, fixation of the percutaneous pedicle screws was done under C-arm guidance. Then, the incision was closed, and a drain was placed (Fig. 1).

### Data collection

Demographic information from all patients was collected, including age, gender, BMI, hypertension, diabetes, surgical level, and follow-up time.

Surgical-related variables including operation time, approach side, visible blood loss, hospital stay, and complications postoperatively were collected after the operation. The operation time was calculated from the beginning of the skin incision to the closure of the incision. The visible blood loss was the sum of estimated blood loss and drainage volume. Since the ULIF was performed under continuous saline irrigation, the estimated blood loss was calculated by the net weight gain of the used surgical gauze added with measuring blood collected by suction canisters and subtracting all irrigation fluids added to the surgical field. The preoperative weight of the dry gauze with exact specifications was known. After the surgery, the circulating nurse weighed the used gauze with an electronic scale. Therefore, we got the net weight gain of the gauze.

VAS and ODI were recorded preoperatively and at 1 and 6 months after operation, and at the last follow-up to evaluate the degree of pain and limb function. The intervertebral fusion was accessed according to the Suk classification [9] through X-ray at the last follow-up and the fusion rate was calculated as follows: Fusion rate = (fusion cases + possible fusion cases)/total cases.

### Statistical analysis

SPSS 26.0 (IBM corporation, USA) was used to perform statistical analysis. Continuous variables are presented as the mean ± standard deviation for normally distributed variables or median with interquartile range for non-normally distributed variables. Enumeration variables were analyzed by the Chi-square test or Fisher’s exact probability test, and quantitative variables were analyzed by Student’s t-test or the Mann–Whitney U-test. Differences were considered statistically significant at a *P* value of < 0.05.

The learning curve of ULIF was analyzed using the *CUSUM* and *RA-CUSUM* methods. The cases were ordered chronologically from the earliest to the last case. The *CUSUM* was based on operation time and the formula was defined as *CUSUM* = $\sum_{i=1}^{n}$*(Xi–U)*, where *Xi* indicates the operation time of each case, *U* indicates the mean operation time of all cases, and *n* represents the consecutive case number. GraphPad Prism 8.0 software was used to plot the results of the *CUSUM* analysis into a scatter diagram, and the function formula was calculated by fitting curve. The *P*-value of less than 0.05 indicates that the fitting curve was successful. The degree of the fitting curve was determined by R^2^, and the closer R^2^ was to 1, the better the curve was fitted. The peak of the fitting curve was obtained by calculating the slope of the curve, thus dividing the learning stage.

The *RA-CUSUM* formula was defined as *RA-CUSUM* = $\sum_{i=1}^{n} (Xi-\zeta )+{(-1)}^{{x}_{i}}Pi$*, Xi* = 1 indicates the presence of surgical failure; *Xi* = 0 indicates the surgery was successful. ζ indicates the probability of actual surgical failure in this study, and *Pi* indicates the expected probability of surgical failure in each case, which was predicted by the multivariate logistic regression model. In this study, surgical failure was defined as the occurrence of complications postoperatively, including dural tears, residual symptoms, epidural hematoma, nerve root injury, wound infection, and cage subsidence. The fitting curve was made based on the results of *RA-CUSUM*, and different learning phases were compared.

## Results

### Demographic characteristics

A total of 184 consecutive patients who underwent single-level ULIF were included in this study. There were 104 males and 80 females, including 49 patients with hypertension and 51 patients with diabetes. The mean age was 65.53 ± 6.21 years, the mean BMI was 23.17 ± 2.45 kg/m^2^, and the mean follow-up time was 12.25 ± 2.49 months. The surgical level was L3/4 in 22 cases, L4/5 in 98 cases, and L5/S1 in 64 cases. Detailed demographic information is shown in Table 1.

**Table 1:** Demographic information

Characteristic	Value
Gender(n)	
Male	104
Female	80
Age(yr)	65.53 ± 6.21
BMI(kg/m^2^)	23.17 ± 2.45
Hypertension(n)	49
Diabetes(n)	51
Surgical segment(n)	
L3/4	22
L4/5	98
L5/S1	64
Follow-up time(mons)	12.25 ± 2.49

### Surgical outcomes

**Fig. 2:**
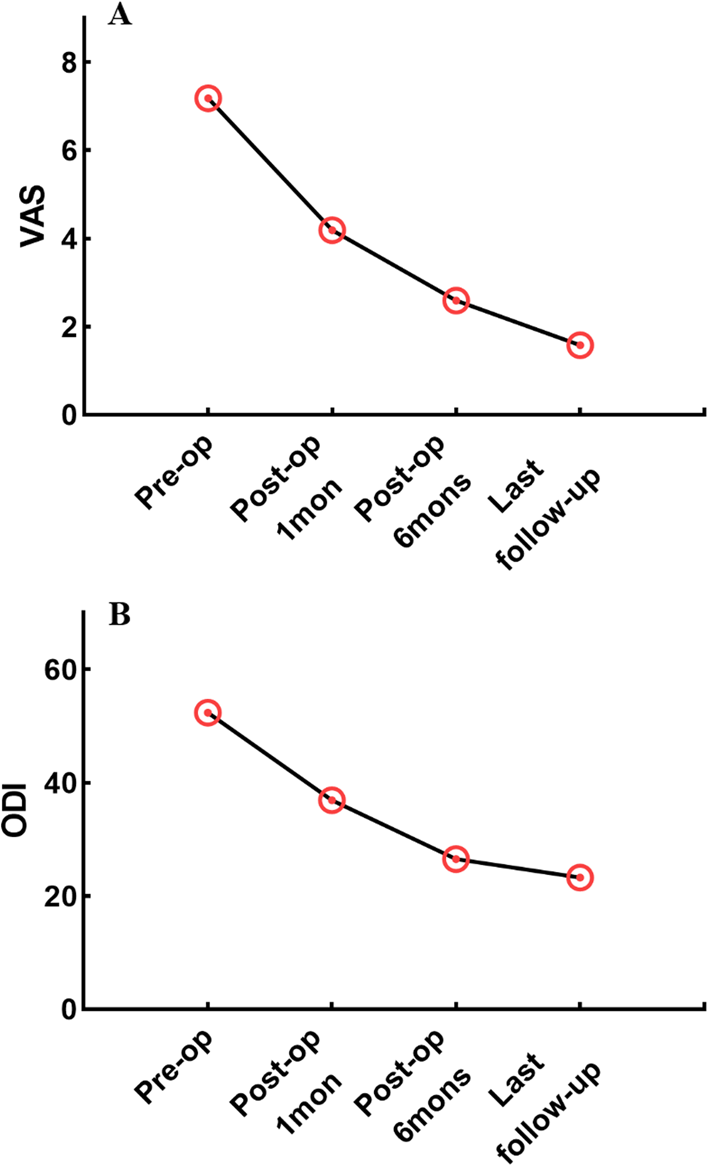
VAS **A** and ODI **B** at different follow-up time points

The mean operation time was 140.14 ± 29.13 mins and the mean hospital stay was 9.39 ± 2.15 days. There were 80 cases with the left surgical approach and 104 cases with the right surgical approach. The mean visible blood loss was 164.80 ± 18.85 ml. The VAS and ODI were significantly improved at the last follow-up compared to those before the operation (*P* < 0.05). At the last follow-up, 155 patients reached intervertebral fusion, 16 were possible intervertebral fusion, and 13 patients failed to reach intervertebral fusion, with a fusion rate of 92.93% (171/184, Table 2, Fig. 2).

**Table 2:** Surgery-related Variable

Variable	Value
Operative time(min)	140.14 ± 29.13
Hospital stay(day)	9.39 ± 2.15
Visible blood loss(ml)	164.80 ± 18.85
Surgical approach(n)	
Left	80
Right	104
Preoperative VAS	7.18 ± 0.85
Postoperative VAS(1 month)	4.19 ± 1.06
Postoperative VAS(6 months)	2.59 ± 0.81
Last follow-up VAS	1.58 ± 0.58
*P* value(VAS)	**0.000**
Preoperative ODI	52.37 ± 3.25
Postoperative ODI(1 month)	36.88 ± 3.09
Postoperative ODI(6 months)	26.50 ± 2.14
Last follow-up ODI	23.23 ± 1.99
*P* value(ODI)	**0.000**
Fusion rate at last follow-up(%, n)	92.9% (171)

Significant values (*P* < 0.05) are in bold

A total of 11 cases were regarded as surgical failure because of complications in this study (Table 3). Three patients had dural tears intraoperatively, and we attempted to use gelatin sponges for compression during the operation and raised the lower limbs of the patients by 30 degrees after the operation. Meanwhile, we closely observed the contents of the drainage sack, and no leakage of cerebrospinal fluid was found. Two cases were found epidural hematomas on postoperative MRI revision. However, the patients did not show any clinical symptoms. Three cases had residual symptoms presenting as no obvious relief of radioactive numbness and pain in the lower limbs, and after intravenous dexamethasone and mannitol injection, the symptoms of the patients disappeared. One case presented with abnormal skin sensation in the innervated area after the operation, which was considered to be nerve root injury, and after conservative treatment, the symptoms disappeared. Two cases at the last follow-up presented with cage subsidence, which was demonstrated as the fusion cage exceeding the upper or lower endplates in the lumbar lateral X-ray.

**Table 3:** Details of complications

Complications	No	No. of cases occurred
Total(n)	11	–
Cage subsidence	2	1st, 11th
Dural tear	3	15th, 20th, 100th
Epidural hematomas	2	17th, 115th
Nerve root injury	1	136th
Residual symptom	3	6th, 27th, 83th

### Learning curve of *CUSUM* analysis

**Fig. 3:**
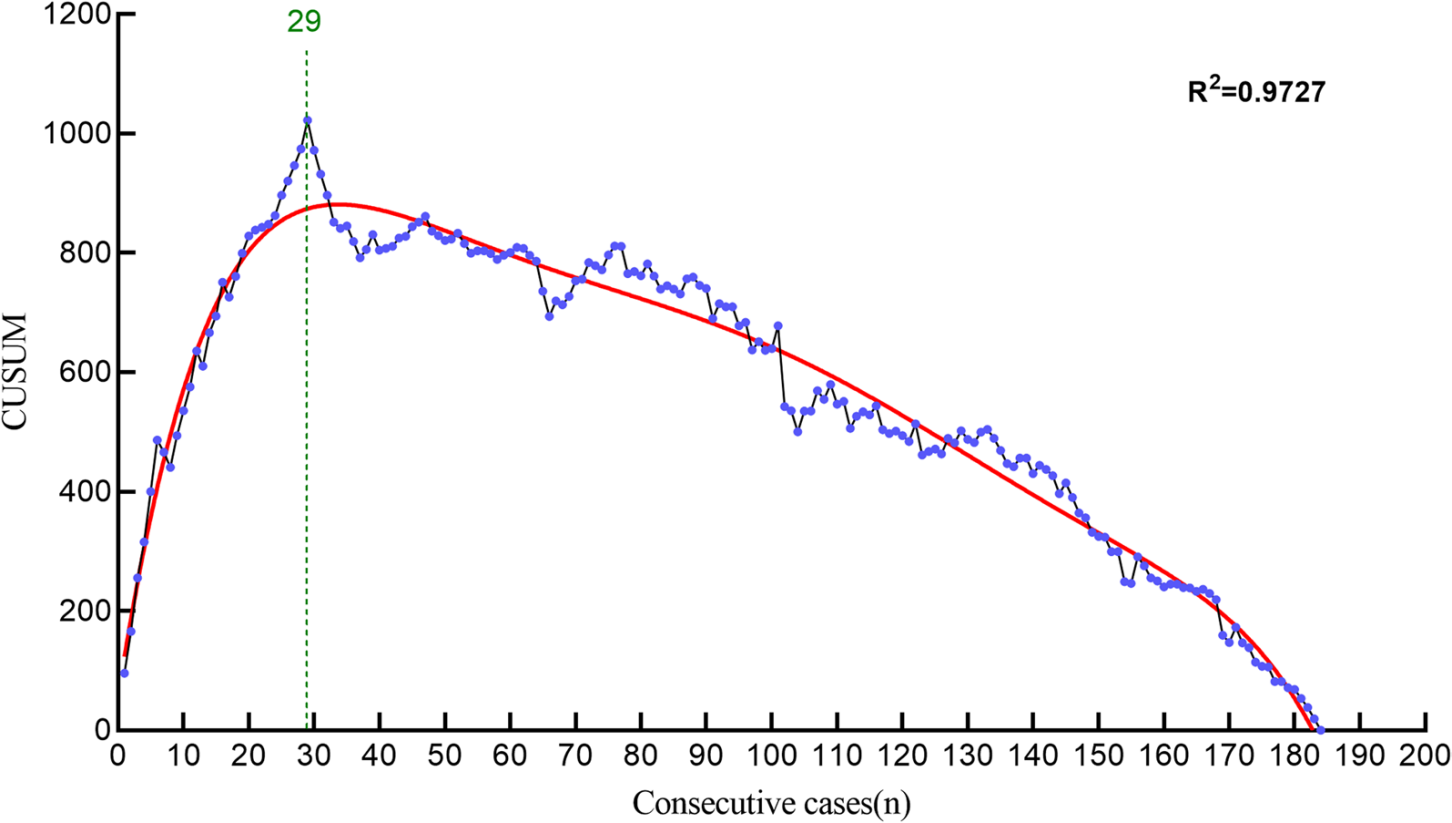
Learning curve of *CUSUM* analysis *CUSUM* = 675.6-4.175 × *n*–0.03848 × *n*^2^–5.653e^−4^ × *n*^3^ + 1.149e^−5^ × *n*^4^ + 1.154e^−7^ × *n*^5^–1.892e^−9^ × *n*^*6*^

The scatterplot was drawn according to the results of *CUSUM*. The fitting curve of the scatterplot gave the function equation as: *CUSUM* = 675.6–4.175 × *n*–0.03848 × *n*^2^–5.653e^−4^ × *n*^3^ + 1.149e^−5^ × *n*^4^ + 1.154e^−7^ × *n*^5^–1.892e^−9^ × *n*^6^(R^2^ = 0.9727, *P* = 0.0000) (Fig. 3). In this study, the slope of the curve changed from positive to negative when crossing the 29th case, indicating that the cutoff point of the fitting curve was 29 cases, which means that the number of cases required for a spine surgeon with UBE experience to become proficient in ULIF was 29 cases. Taking 29 cases as the cutoff point, we divided the learning curve into two phases, the learning phase (1–29 cases) and the mastery phase (30–184 cases). Comparison of patient characteristics and perioperative data at different learning stages are shown in Table 4. The mastery phase showed less operation time and less hospital stay than the learning phase (*P* < 0.05). Additionally, the mastery phase showed fewer surgical failure outcomes (*P* < 0.05). However, there was no significant difference between the two phases in visible blood loss and fusion rate (*P* > 0.05).

**Table 4:** Comparison of different learning phases according to the *CUSUM* analysis

Variable	Total	Phase		*P* value	*x*^2^
	n = 184	Learning Phase(n = 29)	Mastery Phase(n = 155)		
Gender				–	0.804
Male	104	17	87	–	–
Female	80	12	68	–	–
Age(yr)	65.53 ± 6.21	63.86 ± 6.46	65.84 ± 6.14	0.450	–
BMI(kg/m^2^)	23.17 ± 2.45	23.21 ± 3.01	23.16 ± 2.34	0.138	–
Operation time(min)	140.14 ± 29.13	175.38 ± 34.23	133.55 ± 22.76	**0.002**	–
Hospital stay(day)	9.39 ± 2.15	13.07 ± 2.28	8.71 ± 1.23	**0.000**	–
Visible blood loss(ml)	164.80 ± 18.85	177.89 ± 16.83	162.35 ± 18.24	0.538	–
Surgical failure, n(%)	11(5.98)	6(20.69)	5(3.22)	–	**0.000**
Fusion rate(n)	92.9% (171)	86.2% (25)	94.2% (146)	–	0.252

Significant values (*P* < 0.05) are in bold

### Learning curve of *RA-CUSUM* analysis

**Fig. 4:**
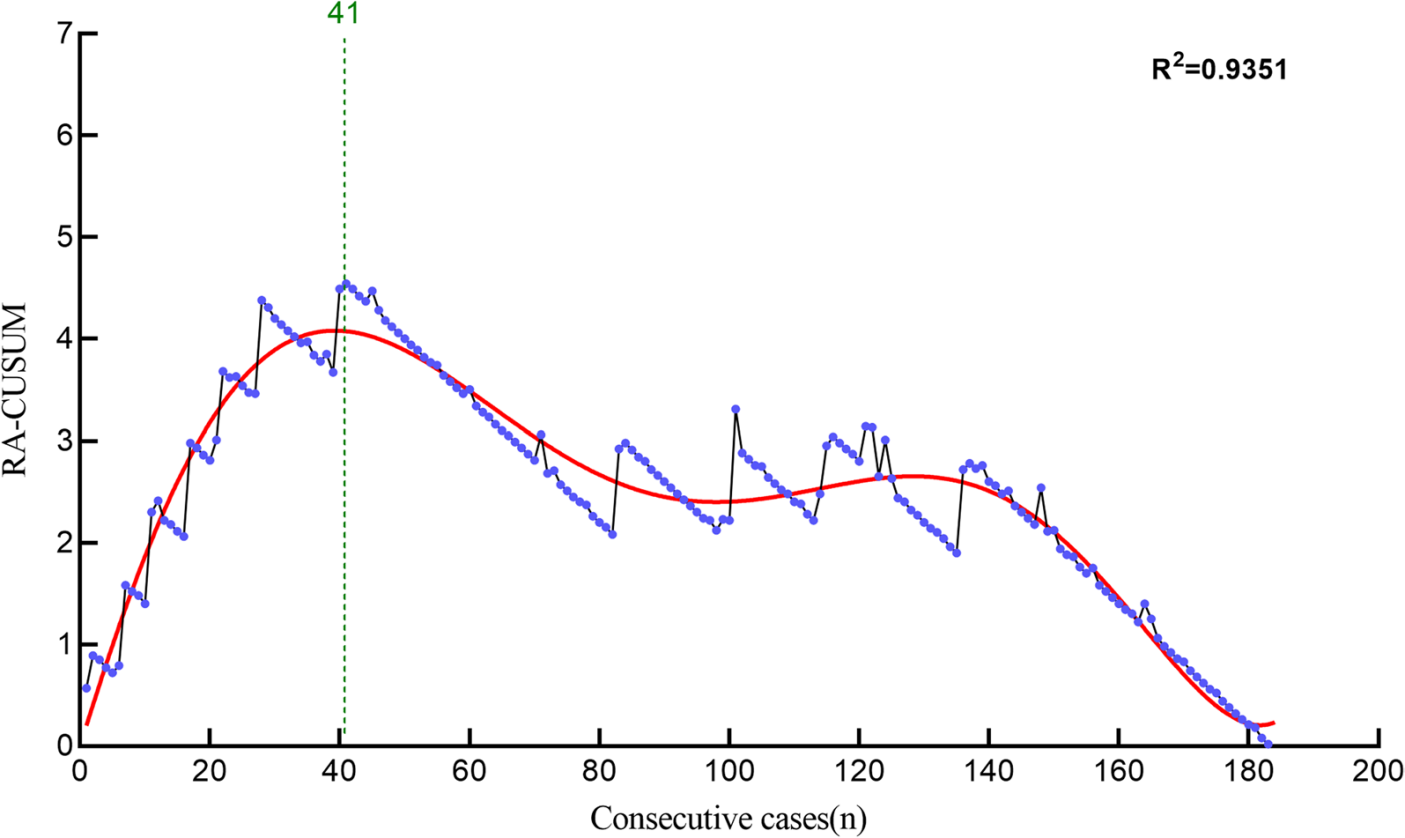
Learning curve of *RA-CUSUM* analysis

Multivariate logistic regression model showed that BMI, hypertension, and operation time were risk factors for surgical failure (*P* < 0.05, Table 5). We obtained the expected probability of surgical failure in each case according to the model predictions, thus obtaining the results of the *RA-CUSUM* analysis. The fitting curve plotted from the results of the *RA-CUSUM* analysis began to show a decrease in slope after the 41st case, indicating that the cutoff point of the fitting curve was 41 cases, which means that a spine surgeon will need to complete 41 cases of ULIF to gradually reduce the probability of surgical failure (Fig. 4). Therefore, the learning curve was divided into a learning phase (1–41 cases) and a mastery phase (42–184 cases). Comparison of demographic and perioperative data between the two learning stages are shown in Table 6. Compared to the learning stage, the mastery stage showed a significant reduction in both operative time and hospital stay (*P* < 0.05). The mastery stage also had fewer probabilities of surgical failure (*P* < 0.05). However, there was no significant difference in fusion rate between the two phases (*P* > 0.05).

**Table 5:** Multivariate Logistic regression of risk factors for surgical failure

Variable	OR/*F*	*P* Value
Univariate *ANOVA* analysis	*F*	
Age	0.579	0.448
Gender	1.304	0.255
BMI	29.198	**0.000**
Hypertension	28.273	**0.000**
Diabetes	0.527	0.469
Operation time	6.854	**0.010**
Surgical segment	3.207	0.075
Visible blood loss	1.936	0.166
Multivariate logistic regression	OR	
BMI	2.095	**0.004**
Hypertension	10.466	**0.048**
Operation time	1.050	**0.005**
Costant	0.000	0.000

Significant values (*P* < 0.05) are in bold

**Table 6:** Comparison of different learning phases according to the *RA-CUSUM* analysis

Variable	Total	Phase		*P* value	*x*^2^
	n = 184	Learning Phase(n = 41)	Mastery Phase(n = 143)		
Gender				–	0.680
Male	104	23	75	–	–
Female	80	18	68	–	–
Age(yr)	65.53 ± 6.21	64.27 ± 6.44	65.88 ± 6.12	0.487	–
BMI(kg/m^2^)	23.17 ± 2.45	23.40 ± 2.73	23.11 ± 2.31	0.482	–
Operation time(min)	140.14 ± 29.13	159.83 ± 39.81	134.50 ± 22.44	**0.000**	–
Hospital stay(day)	9.39 ± 2.15	11.93 ± 2.68	8.67 ± 1.24	**0.000**	–
Visible blood loss(ml)	164.80 ± 18.85	174.00 ± 16.6	162.17 ± 18.68	0.458	–
Surgical failure, n(%)	11(5.98)	7(17.07)	4(2.6)	–	**0.000**
Fusion rate(%, n)	92.9% (171)	85.4% (35)	95.1% (136)	–	0.074

Significant values (*P* < 0.05) are in bold

## Discussion

Lumbar interbody fusion (LIF) is a well-established surgical technique for treating degenerative spinal diseases [10], with advantages in stabilizing painful segments, restoring lumbar lordosis, correcting spinal deformities, and decompressing nerves [11]. However, conventional open lumbar fusion surgery has always been associated with greater tissue damage, more blood loss, and slower postoperative recovery [12], which is detrimental to the patient. To minimize surgical trauma and postoperative complications, spine surgeons are committed to combining minimally invasive concepts with endoscopic techniques. The emergence of spinal endoscopy has enabled spinal surgery to make the leap from open to minimally invasive surgery. Studies have shown that spinal endoscopic lumbar fusion can obtain favorable results in the treatment of degenerative spinal diseases [13]. However endoscopic lumbar interbody fusion (Endo-LIF) is still associated with several limitations. Firstly, the surgical field of Endo-LIF is relatively limited. Because of the limitation of the operating trocar, it is difficult to tilt the instruments, and it is often necessary to tilt the operation table to observe the contralateral lateral recess. These processes will cause unnecessary trouble to the operator, resulting in prolonged operation time [14]. Secondly, because of the limitations of the operation tubular size, it is not possible to place larger cages, which may affect the intervertebral fusion [15]. The UBE technique allows the establishment of portals through the skin without the limitation of operating a trocar, meanwhile, ULIF can place a larger cage and adjust the cage angle more conveniently, which may be the reason why ULIF has better fusion rate [16]. Previous studies have shown that ULIF presents the advantages of less trauma, less bleeding, faster postoperative recovery, and favorable fusion rate [5]. A meta-analysis by Yu et al. [17] indicated that compared to conventional TLIF, ULIF has the advantages in relieving postoperative pain, shortening hospital stay, and enhancing functional recovery. Liu et al. [12] performed a prospective cohort study and found that ULIF has the advantages of minimizing surgical trauma and reducing inflammatory reaction compared to posterior lumbar interbody fusion (PLIF). Our study also found that ULIF showed favorable results in alleviating postoperative pain and improving functional recovery.

The learning curve reflects the speed of mastering skills over a certain time. For beginners, it is usually the number of cases required to reach relative stability in surgical technique [18]. The learning phase of ULIF also requires a lot of clinical experience and a lot of practice. Different from unilateral biportal lumbar discectomy (UBLD), ULIF requires endoscopy insertion into the vertebral body space for endplate preparation and needs to place the cage and adjust the orientation under indirect visualization during operation, which is not exactly similar to open lumbar fusion surgery. Even for spine surgeons with UBE experience, there are still challenges in the early learning phase of ULIF. Chen [19] found that for spine surgeons with no arthroscopic experience, operative time gradually steadied after completing 24 cases of UBLD, suggesting that the surgeons were able to achieve a more proficient and stable performance level. Xu [20] found the significant reduction in operation time for spine surgeons after completing 54 cases of UBLD. After completing 89 cases, the success rate of the procedure began to be stable, suggesting that experience was still required to achieve a higher success rate after overcoming the learning curve. Kim [21] considered that at least 34 cases were needed to master the ULIF. Although the definition of the learning curve is influenced by a variety of subjective factors, surgeon experience, team coordination, surgical instrumentation differences, and differences in operating room procedures may all influence the definition of the learning curve. We can conclude objective and replicable experiences to provide technical references and reduce unnecessary learning time and costs [18].

*CUSUM* was first described by E.S. Page in 1954 and was initially used as monitor performance in the manufacturing industry. Since then, it has been implemented to assess technical training in a variety of procedures [22]. *CUSUM* analysis is an excellent statistical metric to quantitatively assess the learning curve, while the *CUSUM* chart is a precise representation of the temporal relationship between the chronological number of cases performed and a surgeon's ability in a specific surgical task [23]. In this study, the *CUSUM* analysis for the operation time showed that the operation time started to plateau gradually when surgical cases reached 29 cases, operation time in the mastery phase was approximately 40 min shorter than those in the learning phase (133.55 ± 22.76 mins vs. 175.38 ± 34.23 mins). However, operation time cannot be used as the only indicator for evaluating the learning curve, and simply selecting operation time as the definition of the learning curve may lead to bias [24]. It is not only the proficiency of the surgeons that determines the learning curve but also the safety and health interests of the patients. Therefore, we further verify the learning curve of ULIF through *RA-CUSUM* analysis [25]. *RA-CUSUM* takes the surgical failure rate as a parameter. We defined all complications that occurred after ULIF as the occurrence of surgical failure and thus constructed a learning curve based on the surgical failure rate. In this study, the *RA-CUSUM* analysis showed that the success rate of surgery began to stabilize gradually when the surgical cases reached 41 cases. The complication rate was significantly lower in the mastery phase (17.07%) than in the learning phase (2.6%) (*P* < 0.05). Notably, the *CUSUM* analysis-based learning curves showed similar results between the two stages of complication rates. In our study, spine surgeons had some experience with UBLD (no less than 150 cases). Therefore, even at the early stage of the ULIF learning phase, there are more advantages in the coordination of two hands and the stabilization of one hand compared to surgeons without UBE experience. This probably explained why even when faced with the more challenging ULIF in our study, the surgeons still had fewer cases to overcome the learning curve than with the UBLD. In our center, spine surgeons are required to undergo standardized training as well as practice on models and solids before they can perform UBE, and to accumulate experience with at least 90 cases of UBLD before they can perform ULIF, which is a natural learning process [20]. ULIF is based on UBE and traditional open surgery, and performing ULIF without UBE experience is very difficult and may cause a negative impact on the safety of the patients as well as surgical outcomes. There are some suggestions on how to shorten the learning curve. We recommend that beginners should choose easier cases in the early stages, while the right-sided approach may be more comfortable for right-handed people to minimize the difficulty of the practices and overcome the curve of UBLD before proceeding to ULIF. In the early stages, the application of the 0° endoscope allows beginners to adapt more quickly to the UBE technique. Additionally, the spinal canal should be completely explored before the end of the procedure, and the viewing portal and the working portal can be exchanged if necessary to expand the exploration range.

Cage subsidence were observed in two cases (1.08%) in this study, both of which occurred during the learning phase, suggesting that protecting the endplates in the early stage of ULIF was a challenge for spine surgeons. In theory, endplate preparation is the key to lumbar fusion. Damage to the endplate or a reduction in the contact area between the endplate and the cage are possible factors for cage subsidence [26]. Endplate preparation requires the insertion of endoscopy into the intervertebral space and removing the intervertebral discs using curette and disc reamers, which can easily cause bony endplate injuries when the instruments are inserted and removed, especially in patients with narrow intervertebral spaces. Additionally, there is a blind spot between the skin and the endoscope at cage placement, which may also be a factor in endplate injury. For beginners, several points should be considered when preparing endplates. While using endplate curettes to remove cartilaginous endplates, the changes in the endplate should be continually monitored with the endoscope, and the evidence of successful endplate preparation is multiple spots of bleeding from the bone. For the right-handed person, instrumentation in and out of the intervertebral space is best done through the right side of the patient, especially on the level L4/5 or L5/S1, because the working portal is located on the rostral side and on these levels the intervertebral space is tilted caudally. Our study found that BMI was a risk factor for surgical failure, which is similar to previous study [27]. Patients with greater BMI are subjected to greater axial stresses on the cage, which may lead to cage subsidence; therefore, in patients with greater BMI, the length of weight-bearing should be appropriately prolonged with close follow-up.

Dural tears were observed in three cases (1.63%), all of which occurred in the lumbar spinal stenosis. Due to the close attachment between the ligamentum flavum and the dural sac, the dural tears occurred when the ligamentum flavum was violently peeled off using the Kerrison rongeur. Since the tear was small, we used gelatin sponges for compression while maintaining lumbar drainage postoperatively [28]. Distributed in the midline or near the midline surface of the dural sac are meningovertebral ligaments varying in thickness and shape from thin strips to thick sheets, which are capable of connecting the dorsal side of the dural sac with the lamina and ligamentum flavum [29]. Insufficient dissection of this structure may be the primary mechanism of the dural tears. We recommend removing the thin strips between the ligamentum flavum and the dural sac with the bent probe and confirming the detachment before biting off the ligamentum flavum. Furthermore, laminectomy that is wide enough to expose the cephalic and caudal edges of the ligamentum flavum, and removal of the ligamentum flavum en bloc, will also help to minimize injury.

Epidural hematoma were observed in two cases (1.08%), however, the two cases did not have any symptoms, which was called asymptomatic epidural hematoma. In patients undergoing lumbar spine surgery without drainage, MRI can detect up to 89% of asymptomatic epidural hematoma [30], whereas the incidence of symptomatic epidural hematomas is less than 1% [31, 32]. Although most cases with epidural hematoma are not accompanied by any clinical symptoms, we still recommend aggressive MRI for early exclusion of symptomatic epidural hematoma in patients presenting with symptoms of postoperative nerve injury. Our study found that hypertension was the risk factor for surgical failure. In patients with suboptimal vascular conditions, blood pressure elevation will be more significant at the end of anesthesia, which will lead to unpredictable bleeding [33], and even epidural hematoma. Studies have shown that [34, 35] using drainage after lumbar surgery significantly reduced the incidence of postoperative asymptomatic epidural hematoma, and we similarly suggest that drainage should be used after ULIF regardless of the bleeding volume. Notably, surgical bleeding may lead to the occurrence of epidural hematoma [31]. The depth of the anesthesia of the patients may influence the pressure of the spinal canal and the blood loss [36], thus from the beginning of the procedure the physician should monitor the anesthesia and provide appropriate control of the depth of the anesthesia. Continuous intraoperative saline irrigation has advantages in controlling bleeding. Keeping the saline level 50–60 cm above the plane of the surgical incision and maintaining the water pressure at 25–30 mmHg can keep a clear surgical field while controlling bleeding better.

There are still some limitations in our study. First, this was a retrospective study, and all surgeries were performed by the same spine surgeon, which is potentially biased. Therefore our experience is not applicable to other spine surgeons. Due to a variety of practical factors, other spine surgeons may overcome the learning curve earlier or later than we did in our case. Second, the spine surgeon in this study had prior experience performing the UBE technique. Considering that all spine surgeons at our center are required to perform UBLD before ULIF, we do not have sufficient data to further analyze the learning curve of ULIF for surgeons lacking UBE experience. Future large-sample, multicenter, prospective studies are still needed for further verification.

## Conclusion

In this study, the learning curve of ULIF was analyzed using *CUSUM* and *RA-CUSUM* analysis based on the operation time and surgical failure rate, respectively. Outcomes demonstrated that after completion of 29 cases of ULIF, operation time gradually steadied. After completing 41 cases of ULIF, the surgical success rate stabilized. Suitable case selection and standardized training can help to shorten the learning curve.
